# Sigma's Non-specific Protease Activity Assay - Casein as a Substrate

**DOI:** 10.3791/899

**Published:** 2008-09-17

**Authors:** Carrie Cupp-Enyard

**Affiliations:** Sigma Aldrich

## Abstract

Proteases break peptide bonds. In the lab, it is often necessary to measure and/or compare the activity of proteases.  Sigma's non-specific protease activity assay may be used as a standardized procedure to determine the activity of proteases, which is what we do during our quality control procedures. In this assay, casein acts as a substrate. When the protease we are testing digests casein, the amino acid tyrosine is liberated along with other amino acids and peptide fragments.  Folin and Ciocalteus Phenol, or Folin's reagent primarily reacts with free tyrosine to produce a blue colored chromophore, which is quantifiable and measured as an absorbance value on the spectrophotometer.  The more tyrosine that is released from casein, the more the chromophores are generated and the stronger the activity of the protease. Absorbance values generated by the activity of the protease are compared to a standard curve, which is generated by reacting known quantities of tyrosine with the F-C reagent to correlate changes in absorbance with the amount of tyrosine in micromoles.  From the standard curve the activity of protease samples can be determined in terms of Units, which is the amount in micromoles of tyrosine equivalents released from casein per minute.

To view this article in Chinese, click here

**Figure:**
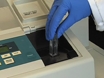


## Protocol

### Before beginning the assay, we need to make sure that the following reagents are correctly prepared:

A 50 mM Potassium Phosphate Buffer, pH 7.5. Prepare using 11.4 mg/ml of potassium phospate dibasic, trihydrate in purified water and adjusting pH with 1M HCl. This solution is placed at 37°C prior to use.
A 0.65% weight/volume casein solution, prepared by mixing 6.5 mg/ml of casein in the 50 mM potassium phosphate buffer. Gradually increased the solution temperature with gentle stirring to 80-85 °C for about 10 minutes until a homogenous dispersion is achieved. It is very important not to boil the solution. The pH is then adjusted if necessary with NaOH and HCl.
A 110 mM Trichloroacetic acid solution, prepared by diluting a 6.1N stock 1:55 with purified water. Trichloroacetic acid is a strong acid and should be handled with care.
0.5 mM Folin & Ciolcaltea's, or Folin's Phenol Reagent, which is the solution that will react with tyrosine to generate a measurable color change that will be directly related to the activity of proteases. Folin's Phenol Reagent is an acid and should be handled with care.
A 500 mM Sodium Carbonate solution, prepared using 53 mg/ml of anyhydrous sodium carbonate in purified water. An enzyme diluent solution, which consists of 10 mM Sodium Acetate Buffer with 5mM Calcium Acetate, pH 7.5, at 37°C. This solution is what we use to dissolve solid protease samples or dilute enzyme solutions.
1.1 mM L-tyrosine Standard stock solution. Prepared using 0.2 mg/ml L-tyrosine in purified water and heated gently until the tyrosine dissolves. As with the casein, do not boil this solution. Allow the L-tyrosine standard to cool to room temperature. This solution will be diluted further to make our standard curve.
Protease solution. Immediately before use, dissolve protease in enzyme diluent solution prepared in step 6.

If necessary, use a solid protease sample of predetermined activity, which is dissolved using enzyme diluent to 0.1-0.2 units/ml. This solution serves as a positive control for the quality control assay and as validation for the calculations we will perform to determine enzyme activity.

### Setting up the Protease Assay and Standard Curves

To begin this assay, find suitable vials that will hold about 15 mls. For each enzyme that will be tested, 4 vials are needed. One vial will be used as a blank, and three others will be used to assay activity of three dilutions of the protease. Three dilutions are useful when checking final calculations against each other.
  To each set of four vials, add 5mls of our 0.65% casein solution. Let them equilibrate in a water bath at 37°C for about 5 minutes.
  Add varying volumes of enzyme solution that will be tested to three of the test sample vials, but not the blank. Mix by swirling and incubate for 37°C for exactly ten minutes. The protease activity and consequential liberation of tyrosine during this incubation time is what will be measured and compared between test samples.
After this 10 minute incubation, add the 5 mls of the TCA reagent to each tube to stop the reaction. Then, add an appropriate volume of enzyme solution to each tube, even the blank, so that the final volume of enzyme solution in each tube is 1 ml. This is done to account for the absorbance value of the enzyme itself and to ensure that the final volume in each tube is equal. Incubate the solutions at 37°C for 30 minutes.During this 30 minute incubation, you may want to set up your tyrosine standard dilutions. Use 6 dram vials (dram vials can be substituted with polypropylene tubes) that can easily hold 8 mls. To the six vials, add the 1.1 mM tyrosine standard stock solutions with the following volumes in mls: 0.05, 0.10, 0.20, 0.40, 0.50. Don't add any tyrosine standard to the blank. Lower standards may be needed for impure test samples that will yield little color change. Once the tyrosine standard solution has been added, add an appropriate volume of purified water to each of the standards to bring the volume to 2 mls.
After the 30 minute incubation, filter each of the test solutions and the blank using a 0.45 um polyethersulfone syringe filter. Filtration is required to remove any insolubles from the samples.
  Add the filtration 2 mls of the test samples and blank filtrate to 4 dram vials that can hold at least 8 mls. The same type of vial in which the standards were prepared can be used.
  To all of the vials containing the standards and standard blank, add 5mls of sodium carbonate. For best results, add 1 ml of Folin's reagent immediately afterwards.
  Add sodium carbonate to regulate any pH drop created by the addition of the Folin's reagent.
  Add sodium carbonate to the test samples and test blank. These solutions become cloudy after the addition of sodium carbonate. Add the Folin's reagent, which will react primarily with free tyrosine.
  Mix the dram vials by swirling and incubate at 37°C for 30 minutes.
  After this incubation, you should notice that the standards have a gradation of color correlating with the amount of tyrosine added; the highest concentrations of tyrosine appearing darkest. You can also notice appreciable color change in our test samples. 2mls of these solutions are filtered using a 0.45 um polyethersulfone syringe filter into suitable cuvettes. Now that the assay is performed, you can proceed to the spectrophotometer to record our absorbance values.

### Measuring Absorbance and Calculating Enzyme Activity

The absorbance of the samples is measured by a spectrophotometer using a wavelength of 660nm. The light path is set to 1cm. Record the absorbance values for the standards, standard blank, the different test samples, and test blank. Once all of the data has been collected, the standard curve can be created. In order to generate the curve, difference in absorbance between the standard and standard blank must be calculated. This is the absorbance value attributable to the amount of tyrosine in the standard solutions. After this simple calculation, create the standard curve using a graphing program to plot the change in absorbance of our standards on the Y axis, versus the amount in micromoles for each of our 5 standards on the X axis.

**Table:** 

Volume of Tyrosine Standard	uMoles Tyrosine
0.05	0.055
0.10	0.111
0.20	0.221
0.40	0.442
0.50	0.553
After data points have been entered, generate a line of best fit and corresponding slope equation.Find the change in absorbance in the test samples by calculating the difference between the test sample absorbance and the absorbance of the test blank. Inserting the absorbance value for one of the test samples into the slope equation and solving will result in the micromoles of tyrosine liberated during this particular proteolytic reaction. To get the activity of enzyme in units per/ml, perform the following calculation:***(umole tyrosine equivalents released) x (11)Units/ml Enzyme = __________________________________________(1) x (10) x (2)11= Total volume (in milliliters) of assay10= Time of assay (in minutes) as per the Unit definition1= Volume of Enzyme (in milliliters) of enzyme used2= Volume (in milliliters) used in Colorimetric Determination***Take the number of micromoles tyrosine equivalents released obtained from the slope equation and multiply it by the total volume of the assay in mls, which in our case is 11mls. Divide this value by three other quantities: the time of the assay, which we ran for 10 minutes, the volume of enzyme used in the assay, which was varied (let's use 1ml), the volume of milliliters used in colorimetric detection, which may differ based on your cuvette. We used 2 mls.
Micromoles of tyrosine divided by time in minutes yields measurement of protease activity called "units". We can cancel out the units for volume measurement in the numerator and denominator, leaving a measurment of enzyme activity in terms of units/ml. In order to determine the activity in a solid protease sample diluted in enzyme diluent, we divide our activity in units/ml by the concentration of solid used in this assay originally in mg/ml., leaving us with activity in terms of units/mg.***Units/ml enzymeUnits/mg solid = _____________________mg solid/ ml enzyme***

## Discussion

We've just shown you how to analyze protease activity using Sigma's universal  protease activity assay. In addition, this assay is useful to ensure that our proteases have precisely determined activity before you receive them for your experiments. As you have seen, when doing this procedure, it's of paramount importance to remember to heat both the casein and tyrosine solutions slowly and not to boil them.  Also, it's critical to prepare different blanks for both your standards and for each test sample that you have.
